# Coronary computed tomography angiography and [^15^O]H_2_O positron emission tomography perfusion imaging for the assessment of coronary artery disease

**DOI:** 10.1007/s12471-020-01445-7

**Published:** 2020-08-11

**Authors:** P. A. van Diemen, S. P. Schumacher, R. S. Driessen, M. J. Bom, W. J. Stuijfzand, H. Everaars, R. W. de Winter, P. G. Raijmakers, A. C. van Rossum, A. Hirsch, I. Danad, P. Knaapen

**Affiliations:** 1 Department of Cardiology, Amsterdam UMC, Vrije Universiteit Amsterdam, Amsterdam, The Netherlands; 2 Department of Radiology, Nuclear Medicine and PET research, Amsterdam UMC, Vrije Universiteit Amsterdam, Amsterdam, The Netherlands; 3 Department of Cardiology and Radiology and Nuclear Medicine, Erasmus MC, University Medical Center Rotterdam, Rotterdam, The Netherlands

**Keywords:** Coronary computed tomography angiography, Positron emission tomography, Myocardial perfusion imaging, Hybrid imaging, Coronary artery disease, Chronic coronary total occlusion

## Abstract

Determining the anatomic severity and extent of coronary artery disease (CAD) by means of coronary computed tomography angiography (CCTA) and its effect on perfusion using myocardial perfusion imaging (MPI) form the pillars of the non-invasive imaging assessment of CAD. This review will 1) focus on CCTA and [^15^O]H_2_O positron emission tomography MPI as stand-alone imaging modalities and their combined use for detecting CAD, 2) highlight some of the lessons learned from the PACIFIC trial (Comparison of Coronary CT Angiography, SPECT, PET, and Hybrid Imaging for Diagnosis of Ischemic Heart Disease Determined by Fractional Flow Reserve (FFR) (NCT01521468)), and 3) discuss the use of [^15^O]H_2_O PET MPI in the clinical work-up of patients with a chronic coronary total occlusion (CTO).

## Dutch contribution to the field

- The Amsterdam UMC, Vrije Universiteit Amsterdam, is one the few sites worldwide that uses [^15^O]H_2_O PET MPI for the assessment of CAD.
- The PACIFIC trial conducted by the Amsterdam UMC, Vrije Universiteit Amsterdam was the first study to compare the diagnostic performance of CCTA, SPECT, [^15^O]H_2_O PET and hybrid imaging in a true head-to-head fashion using FFR as reference standard.
- Numerous PACIFIC trial substudies have contributed to an improved understanding of the assessment of CAD by means of CCTA and [^15^O]H_2_O PET.
- In the dedicated CTO program of the Amsterdam UMC, Vrije Universiteit Amsterdam, [^15^O]H_2_O PET MPI has been employed to assess the presence of ischaemia in patients with a possible indication for percutaneous revascularisation of their CTO.

## Introduction

Coronary atherosclerosis is marked by a chronic inflammation of the coronary arteries leading to accumulation of lipids and inflammatory cells in the arterial wall (plaques) [1]. Development of plaques may take decades but by diminishing blood flow to the subtended myocardium can eventually lead to ischaemia causing symptoms such as chest pain and dyspnoea. It is vital to assess the presence and extent of coronary artery disease (CAD) in patients with suspected CAD in order to determine the correct diagnosis and appropriate treatment strategy [2]. The non-invasive imaging modalities, coronary computed tomography angiography (CCTA) and positron emission tomography (PET) myocardial perfusion imaging (MPI) are widely utilised to that extent and assess the anatomic severity and functional significance of CAD, respectively. In this review we will highlight the assessment of CAD by means of CCTA and [^15^O]H_2_O PET MPI focussing on studies performed by Dutch investigators.

## Coronary computed tomography angiography

**Fig. 1:**
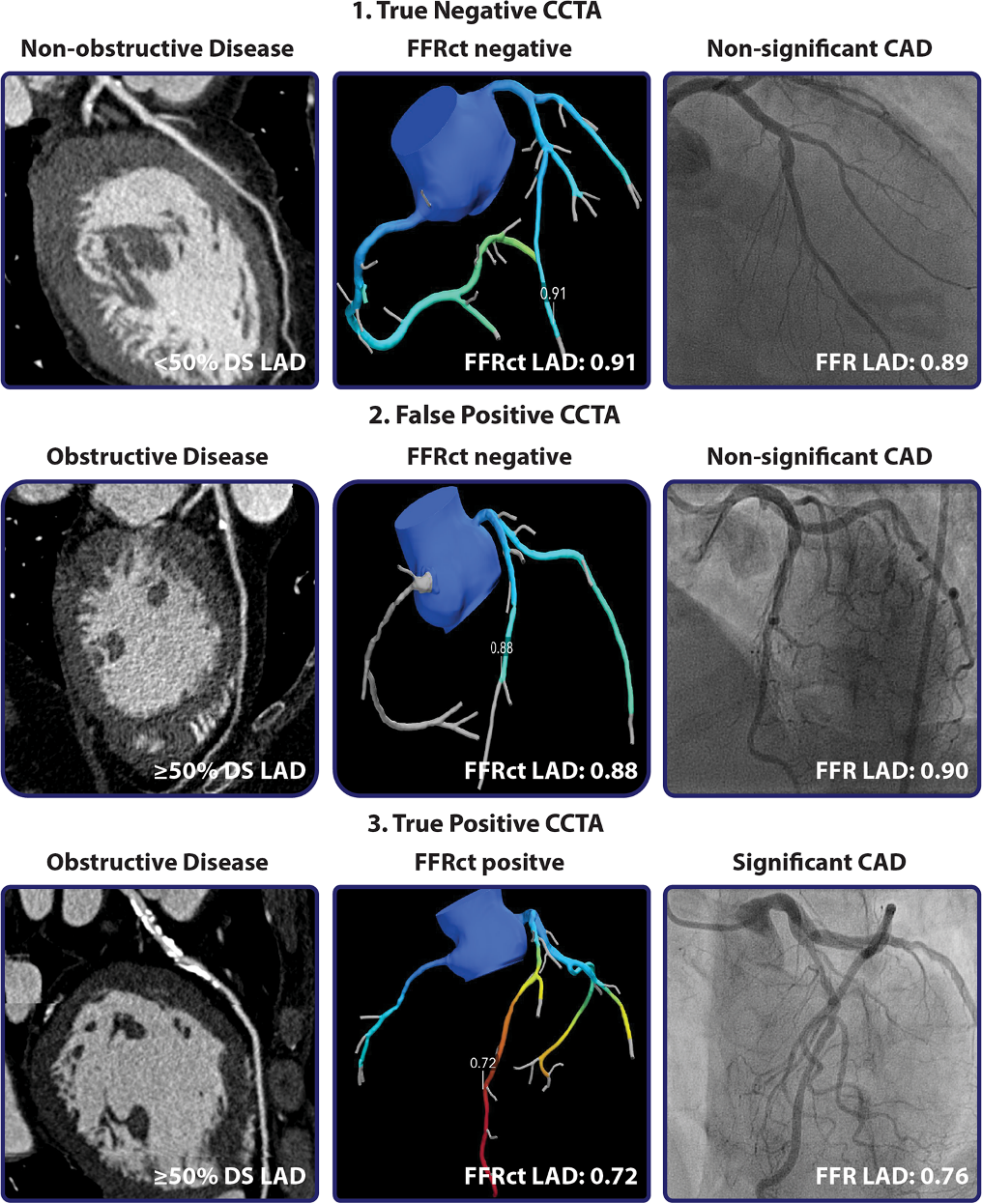
Case examples of CCTA with incorporation of FFRct and the ICA result. Case 1 presents the CCTA of a patient with non-obstructive disease in the LAD, as expected owing to the high sensitivity and negative predictive value of CCTA, subsequent ICA with FFR measurements confirmed non-significant CAD. The diagnostic performance of CCTA is, however, hampered by a relatively high rate of false-positive findings, an example is seen in Case 2. Incorporation of FFRct analysis in the assessment of CCTA can lead to a shift from false-positive results to true negatives (Case 2) and can confirm the significance of CAD as seen in Case 3. *CAD* coronary artery disease, *CCTA* coronary computed tomography angiography, *DS* diameter stenosis, *FFR* fractional flow reserve, *FFRct* CCTA derived FFR, *ICA* invasive coronary angiography, *LAD* left anterior descending artery

CCTA may represent a good alternative for invasive coronary angiography (ICA), especially in patients with a low or intermediate pre-test likelihood of CAD [2]. It is an anatomical imaging modality that allows for the assessment of extent and severity of coronary atherosclerosis. A large body of evidence demonstrates that CCTA is able to exclude significant CAD with a near to absolute certainty due to its excellent sensitivity and negative predictive value [3]. Nevertheless, it is hampered by a high rate of false-positive findings and as such its specificity and positive predictive value is only moderate [3]. This is explained by the tendency of CCTA to overestimate the severity of disease due to artifacts caused by, for example, calcifications, known as ‘blooming artifacts’ (Fig. 1; [4]). Prospective studies have shown that patients who underwent CCTA as a first-line test were more likely to be referred for ICA and even be revascularised as a consequence compared with those who underwent a functional test or standard care [5, 6]. On the other hand, the rate of non-obstructive CAD on ICA following CCTA is also higher as compared with a diagnostic strategy that utilises a functional test [5]. This highlights the limitations of CCTA since the burden of calcification seen on computed tomography does not directly relate to the degree of luminal obstruction, let alone its functional consequences. However, CCTA has justly acquired a prominent place in contemporary guidelines as a first-line test for the evaluation of symptomatic patients with a low to intermediate pre-test likelihood of obstructive CAD [2]. Accordingly, guidelines recommend a functional test in the presence of obstructive CAD on CCTA, known as the hybrid approach, as viable diagnostic strategy in order to minimise the rate of false-positive CCTA findings and as such lead to a more judicious referral for ICA [2]. Recently, a CCTA-based technique has been developed that assesses lesion-specific ischaemia, namely FFRct: fractional flow reserve derived from CCTA [7]. FFRct (HeartFlow Inc. Redwood City, USA) uses computational fluid dynamics and a 3D model of the coronary vasculature derived from standard CCTA datasets to calculate FFR [7]. Prospective trials have consistently demonstrated FFRct to accurately detect lesion-specific ischaemia [3, 8, 9]. The FFRct PACIFIC sub-study was the first study to compare the accuracy of CCTA, FFRct, single-photon emission computed tomography (SPECT), and positron emission tomography (PET) myocardial perfusion imaging (MPI) in a head-to-head manner and demonstrated FFRct to exhibit the highest accuracy for lesion-specific ischaemia as refereed by invasive FFR. Noteworthy, FFRct could not be obtained in 17% of the vessels [10]. Furthermore, incorporating FFRct in CCTA assessment possibly reduces healthcare costs without a penalty to clinical outcome as compared with standard care [11]. Fig. 1 demonstrates how FFRct can lead to a more prudent referral pattern for ICA. Another approach to predict the functional significance of CAD solely based on CCTA is related to parameters of severity and burden of atherosclerosis, such as total plaque volume, non-calcified plaque volume and adverse plaque characteristics that have all been linked to the presence of ischaemia [12–14]. These analyses are, however, time-consuming and therefore not yet applicable in daily practice. Implementation of new technologies such as machine learning may overcome this barrier [15]. Machine learning has the potential to run these analyses swiftly and with high accuracy and consistency. Future studies, such as the CONFIRM-II trial, will investigate whether machine-learning analysis provides improved diagnostic accuracy and prognostication compared with human readers.

## [^15^O]H_2_O PET perfusion imaging

**Fig. 2:**
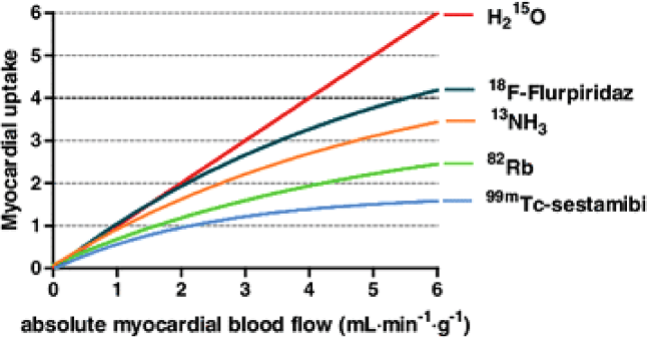
Kinetics of tracers used for PET MPI. Graphical presentation of the relationship between absolute MBF and actual tracer uptake of the PET tracers; [^15^O]H_2_O, ^13^NH_3_, and ^82^RB. ^18^F‑Flurpiridaz is a PET tracer currently being tested in a phase III trial (NCT03028740) and therefore not yet used in clinical practice. ^99m^Tc-sestamibi is the tracer frequently used for single-photon emission computed tomography MPI. Figure adapted from Danad et al. [24]. Adapted from and with permission of Springer. *PET* positron emission tomography, *MPI* myocardial perfusion imaging

**Fig. 3:**
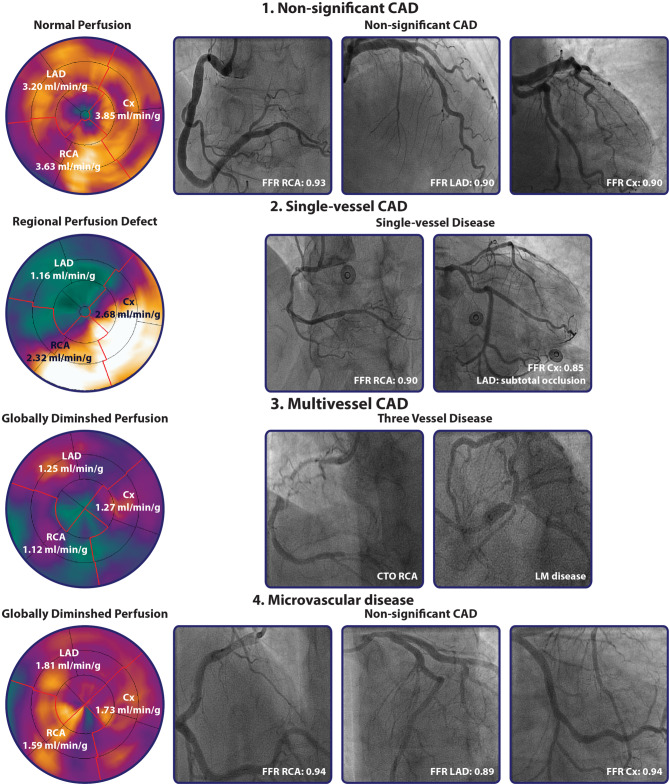
Case examples of [^15^O]H_2_O PET MPI and subsequent ICA. Case examples of results obtained through [^15^O]H_2_O PET MPI and subsequent ICA with FFR measurements. Case 1 demonstrates a patient with normal hyperaemic perfusion above the cut-off defining ischaemia in all vascular territories (≤2.30 ml/min/g), ICA in conjunction with FFR measurements confirmed the presence of non-significant CAD. A defect with diminished hyperaemic perfusion in the LAD territory is displayed in Case 2, the patient was referred for ICA which demonstrated a sub-total lesion of the proximal LAD with non-significant CAD of the RCA and Cx. Furthermore, quantitative PET MPI can be used to determine the presence of globally diminished perfusion, which can be due to multivessel CAD (Case 3) or possible microvascular disease (Case 4). *CTO* chronic coronary total occlusion, *Cx* circumflex artery, *RCA* right coronary artery, other abbreviations as in Figs. 1 and 2

Nuclear-based functional testing is at the heart of diagnosing CAD. For decades, the field of MPI has been dominated by SPECT. From the outset, SPECT has been the MPI workhorse. However, over the last years a switch from SPECT to PET MPI has been taking place, given the increasing availability of PET scanners and ^82^Sr/^82^Rb generators, lower radiation exposure, improved resolution, ability of PET to quantify perfusion in absolute terms (in ml/min/g) and lastly superior pharmacokinetics of the tracers used as compared with SPECT tracers [16]. There is a wide variety of PET perfusion tracers available such as ^82^Rb, ^13^NH_3_ and [^15^O]H_2_O [16, 17]. Nowadays, ^82^Rb is the most widely utilised tracer; however, clinical use of [^15^O]H_2_O is expected to take a leap forward with the completion of a multicentre phase III trial that will evaluate [^15^O]H_2_O PET versus ICA and current best practice SPECT imaging to obtain United States of America (USA) Food and Drug Administration (FDA) approval for [^15^O]H_2_O as a PET tracer in the USA. There are some distinct pharmacokinetic differences between the tracers. Both ^82^Rb and ^13^NH_3_ are transported to and trapped within the myocardium, whereas [^15^O]H_2_O is freely diffusible, metabolically inert and completely extracted from the arterial blood pool by myocardium rendering it an ideal tracer to quantify myocardial blood flow (MBF) in ml/min/g (Fig. 2; [16, 17]). The added value of MBF quantification is that it allows for detection of microvascular disease and three-vessel disease or left main disease, which might go unnoticed on relative uptake images of PET and SPECT as these are dependent on normally perfused myocardium to serve as reference area (Fig. 3; [18, 19]). The optimal quantitative MBF cut-off to detect significant CAD has been studied by Danad and colleagues, who showed a hyperaemic MBF of ≤2.3 ml/min/g to be the optimal threshold to detect FFR-defined disease [20]. In addition to hyperaemic MBF, coronary flow reserve (CFR) can be calculated by dividing hyperaemic MBF by baseline MBF. CFR has a lower accuracy for detecting significant CAD as compared with hyperaemic MBF [20]. Dependency of CFR on both baseline and hyperaemic MBF probably contributes to this finding, as diminished CFR is not necessarily concomitant with reduced hyperaemic MBF but can be a result of high baseline values. Although CFR has been shown to be of incremental prognostic value it seems justified that for diagnostic purposes stress-only PET protocols suffice, obviating the need for baseline perfusion imaging leading to a reduction of radiation dose and scan acquisition time [21, 22]. Furthermore, as recently published, [^15^O]H_2_O PET derived hyperaemic MBF predicts adverse patient outcome independently of CFR in patients with suspected CAD [23].

## Hybrid cardiac PET/CCTA imaging, more than the sum of its parts?

Interestingly, [^15^O]H_2_O PET can be performed on hybrid PET/CT scanners which allow assessment of coronary anatomy and functional significance of observed disease within one single scanning session [24]. In the Amsterdam University Medical Center (UMC), a clinical cohort of patients with suspected obstructive CAD underwent combined CCTA and [^15^O]H_2_O PET MPI as part of their diagnostic work-up. Among these patients a hybrid approach led to a higher diagnostic certainty as compared with either modality alone, mainly by reducing the rate of false-positive CCTA findings [25]. Furthermore, hybrid PET/CCTA imaging could impact clinical decision-making, wherein MPI served as a valuable gatekeeper leading to less referral of patients for ICA when an abnormal or equivocal CCTA outcome was observed [26]. However, the true additive value of hybrid imaging remained debated due to the retrospective nature and lack of an appropriate reference standard of the aforementioned studies. As such, the PACIFIC trial was designed to determine whether stand-alone anatomic assessment by CCTA or stand-alone functional assessment by SPECT or PET MPI was superior in terms of diagnostic accuracy and if a hybrid approach provided incremental diagnostic value [27]. A total of 208 patients with suspected CAD without a cardiac history underwent CCTA, SPECT, and PET in a true head-to-head fashion followed by ICA in conjunction with interrogation of all major coronary arteries by invasive FFR regardless of imaging findings and stenosis severity. The diagnostic performance of CCTA, SPECT, and PET when refereed by FFR measurements is displayed in Tab. 1. In summary, quantitative [^15^O]H_2_O PET exhibited a significantly higher accuracy as compared with CCTA and SPECT. In addition, CCTA proved to be an ideal tool for the exclusion of significant CAD as reflected by its high sensitivity and negative predictive value. An important finding was the unexpectedly low sensitivity of SPECT as a result of a high number of false-negative findings. The putative accuracy of SPECT derived from earlier studies is controversial due to the use of an anatomical reference standard, namely obstructive disease on ICA [28]. Furthermore, the unfavourable pharmacokinetics of SPECT tracers led to a high rate of false-negative findings when referenced by FFR (Fig. 2; [16]). The addition of functional testing to CCTA increased specificity by reducing the number of false-positive CCTA findings but came with a penalty to sensitivity as a result of false-negative MPI results [27]. As such, there is paradoxically no incremental diagnostic value of combining MPI with CCTA. The findings of the PACIFIC trial have been confirmed by the prospective Danish Study of Non-Invasive Diagnostic Testing in Coronary Artery Disease (Dan-NICAD) showing a low sensitivity of SPECT (36%) and cardiac magnetic resonance imaging (41%) MPI in patients with obstructive CAD on CCTA [29]. Interestingly, both studies have in common that FFR was used as reference standard instead of obstructive disease on ICA. A multitude of sub-studies utilised the [^15^O]H_2_O PET and CCTA data obtained in the PACIFIC trial of which we will highlight a few.

**Table 1:** Diagnostic performance of CCTA, SPECT, [^15^O]H_2_O PET, and hybrid imaging for diagnosing FFR-defined significant CAD as observed in the PACIFIC trial [27]. Adapted from and wth permssion of the American Medical Association

	% (95% confidence interval)				
Characteristics	CCTA	SPECT	PET	SPECT/CCTA	PET/CCTA
*Per patient*					
Sensitivity	90 (82–95)	57 (46–67)	87 (78–93)	50 (39–61)	74 (64–83)
Specificity	60 (51–69)	94 (88–98)	84 (75–89)	97 (93–99)	92 (86–96)
PPV	64 (55–73)	88 (77–95)	81 (72–89)	94 (83–99)	88 (79–94)
NPV	89 (80–95)	73 (65–80)	89 (81–94)	71 (63–78)	82 (74–88)
Accuracy	74 (67–79)	77 (71–83)	85 (80–90)	76 (70–82)	84 (79–89)
*Per vessel*					
Sensitivity	72 (64–79)	39 (32–48)	81 (73–87)	35 (27–43)	64 (55–71)
Specificity	78 (74–82)	96 (94–98)	75 (69–81)	99 (98–100)	97 (95–98)
PPV	52 (44–59)	80 (70–87)	59 (51–66)	87 (65–96)	87 (79–92)
NPV	87 (83–91)	81 (76–85)	92 (88–95)	81 (76–85)	88 (84–91)
Accuracy	77 (73–80)	82 (78–85)	79 (75–83)	83 (79–86)	88 (85–91)

Table adapted from Danad et al. [27]

*CCTA* coronary computed tomography angiography, *NPV* negative predictive value, *PET* positron emission tomography, *PPV* positive predictive value, *SPECT* single-photon emission computed tomography

## CCTA derived plaque burden and morphology, more than meets the eye

**Fig. 4:**
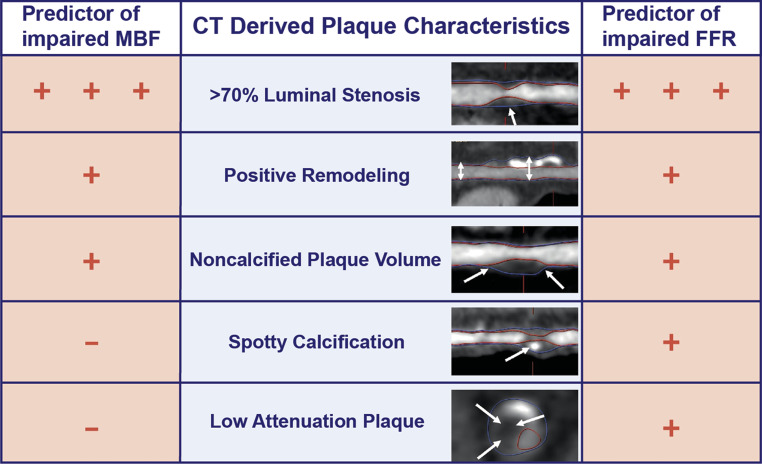
The association of CCTA derived plaque characteristics with impaired hyperaemic MBF measured by [^15^O]H_2_O PET and invasively measured FFR. Driessen et al. studied the effect of CT-derived plaque characteristics on hyperaemic MBF and FFR and demonstrated luminal stenosis severity to be the strongest predictor of impaired hyperaemic MBF and FFR. Positive remodelling and noncalcified plaque volume negatively influenced perfusion and FFR, whereas spotty calcification and low attenuation plaque affected FFR but not hyperaemic MBF. Figure adapted from Driessen et al. [12]. Adapted from and with permission of Elsevier. *MBF* myocardial blood flow, other abbreviations as in Figs. 1 and 2

As mentioned previously, CCTA allows for the assessment of obstructive CAD and in addition permits the visualisation and quantification of plaque burden and morphology. Adverse plaque characteristics such as positive remodelling, low attenuation plaque, and spotty calcification are associated with the occurrence of acute coronary syndromes [30, 31]. Plaque burden and morphology harbours, beside prognostic value, information about the effect of atherosclerosis on downstream perfusion as assessed by [^15^O]H_2_O PET and FFR (Fig. 4; [12]). Driessen et al. showed positive remodelling and non-calcified plaque volume to have a detrimental effect on both hyperaemic MBF and FFR independent of lesion severity, whereas spotty calcification and low attenuation plaque negatively affected FFR but not [^15^O]H_2_O PET derived hyperaemic MBF [12]. In contrast to FFR, the invasively obtained resting pressure index instantaneous wave-free ratio (iFR) showed not to be associated with high-risk plaque features [32].

## Reversing the roles: invasively measured indices referenced by [^15^O]H_2_O PET determined MBF

As mentioned previously, [^15^O]H_2_O PET derived MBF is considered the reference standard for non-invasive assessment of quantitative myocardial perfusion. However, absolute coronary flow can also be invasively measured using continuous intracoronary infusion of saline, known as continuous thermodilution. Everaars et al. were the first to validate the invasive quantification of MBF by means of this thermodilution technique using [^15^O]H_2_O PET derived MBF as reference and demonstrated a near perfect correlation between the two indices [33]. This novel technique is, however, not yet used in clinical practice in contrast to routinely obtained pressure indices FFR, iFR and ratio of resting distal pressure (Pd) and aortic pressure (Pa) (Pd/Pa), which are all able to assess the functional significance of epicardial lesions [34]. Whereas FFR is measured during hyperaemic conditions, iFR and resting Pd/Pa are obtained without inducing hyperaemia. De Waard et al. investigated whether resting invasive pressure indices were capable of detecting impaired hyperaemic MBF as well as the invasive reference standard FFR, and demonstrated all pressure indices to have a similar diagnostic performance when referenced by [^15^O]H_2_O PET. This supports the invasive functional assessment of CAD during resting conditions [34].

## Do we need MPI in the future or can computational models do the job?

In recent years novel techniques have been developed that assess lesion-specific significance by estimating invasive FFR solely based on 3D models of the coronary vasculature and computational fluid dynamics. Advantages of these computational models are that they obviate the need to use pressure wires and induce hyperaemia. One of these techniques is FFRct, which was highlighted previously, another is quantitative flow ratio (QFR) which is derived from ICA cine contrast images. FFRct and QFR demonstrate a similar and high diagnostic accuracy when referenced by FFR [3, 35]. In the PACIFIC population, QFR had a higher accuracy compared with SPECT and PET MPI for the diagnosis of lesion-specific ischaemia [36]. Noteworthy, QFR computation was not feasible in 48% of the vessels due to the lack of a predefined dedicated QFR acquisition protocol in the PACIFIC trial hampering a per-patient analysis. Introduction of these computational-based techniques in the clinical arena will delineate their role in the diagnostic armamentarium.

## [^15^O]H_2_O PET MPI in patients with chronic coronary total occlusion

Clinical guidelines emphasise the importance of ischaemia and viability assessment in patients with a chronic coronary total occlusion (CTO) prior to revascularisation due to the slightly increased risk of procedural complications as compared with revascularisation of non-CTO lesions and furthermore to establish an appropriate indication [37]. In the dedicated CTO program of the Amsterdam UMC, [^15^O]H_2_O PET MPI is used to assess the presence and extent of ischaemia in patients with a potential indication for percutaneous coronary intervention (PCI) of a CTO. Prior reports from this program demonstrated marked ischaemia (>10% of the left ventricle) to be present in practically all patients with a CTO irrespective of collateral status [38, 39]. In fact, the median extent of ischaemia related to the CTO lesion was 24% of the left ventricle [39]. Of note, all patients had an indication for evaluation of the CTO with the majority of patients (>80%) being symptomatic. Furthermore, the extent and depth of ischaemia was observed to be more profound in patients with a CTO as compared with patients with severe haemodynamically significant lesions as determined by FFR (mean FFR: 0.55 ± 0.19) [10, 40]. These findings may be expected given the absence of antegrade flow and the complete dependence of myocardium subtended by a CTO on collateral supply. However, in clinical practice it is regularly assumed that well-developed collaterals preclude stress-induced ischaemia. This assumption may be refuted and should not be used as a reason to defer a patient from revascularisation.

## [^15^O]H_2_O PET MPI to evaluate effects of CTO PCI

**Fig. 5:**
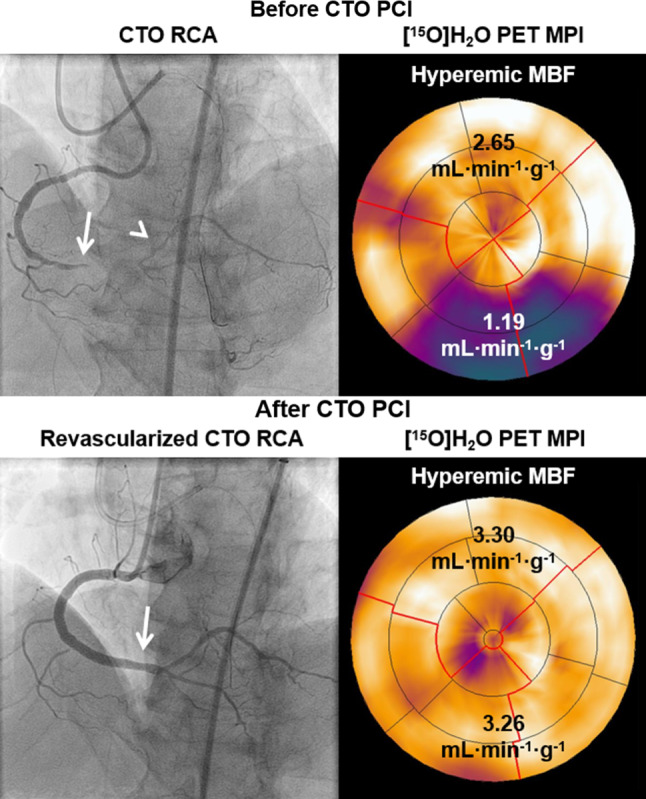
A [^15^O]H_2_O PET MPI case example of recovery of absolute myocardial perfusion after successful CTO PCI. Before PCI, a reduced hyperaemic MBF was observed with [^15^O]H_2_O PET MPI in myocardium subtended by a CTO in the distal RCA (arrow shows the proximal cap) despite the presence of collaterals arising from the left coronary artery supplying the distal vascular territory (arrowhead) of the CTO. Note that the collaterals are not clearly visible due to prolonged filming to get a clear view of the lesion’s distal cap. Successful CTO PCI resulted in restoration of antegrade blood flow and normalisation of hyperaemic MBF which was reassessed 3 months after revascularisation. *MBF* myocardial blood flow, *PCI* percutaneous coronary intervention, other abbreviations as in Figs. 2, 3 and 4

Patients treated successfully by CTO PCI in the Amsterdam UMC were prospectively rescheduled for [^15^O]H_2_O PET MPI 3 months after revascularisation to evaluate the effects on myocardial perfusion. Stuijfzand et al. demonstrated that CTO PCI resulted in large reductions of the perfusion defect size accompanied by significant increases in hyperaemic MBF (Fig. 5; [38]). The median decrease in defect size after CTO PCI was reported to be three segments which equals 17.5% of left ventricular myocardium according to the standardised 17-segment model and can be considered a substantial reduction in ischaemic burden [39, 41]. In addition, successful CTO PCI improved myocardial perfusion to a similar extent as successful PCI of haemodynamically significant non-occlusive lesions in a subgroup of patients from the PACIFIC trial [10, 39, 41]. These results indicate that the expected benefit of CTO PCI, if successfully and safely performed by experienced hands, should not be considered inferior to non-CTO PCI if (silent) ischaemia reduction is the indication for revascularisation. Of note, microvascular (dys)function has an important impact on the ability to restore perfusion. Several risk factors for microvascular dysfunction (left ventricular dysfunction, a history of myocardial infarction in the CTO territory) are negative predictors of improvement in hyperaemic MBF [42]. In contrast, if hyperaemic MBF is higher in surrounding myocardium not subtended by obstructive CAD (indicating normal functioning microvasculature), the gain in hyperaemic MBF in the CTO area that can be expected after PCI is higher as well [42].

## Conclusion

Coronary CTA and MPI are established non-invasive imaging modalities to diagnose CAD with technique-dependent advantages such as the high negative predictive value of CCTA and the ability of MPI to assess the functional severity of CAD. Computational fluid-based techniques such as FFRct and QFR diversify the diagnostic opportunities available to the physician. Although novel insights and developments in the field of (non)invasive imaging are promising and might lead to a more judicious assessment of CAD, the incremental value of imaging-based treatment strategies to improve patient outcome should be carefully reviewed.
